# Organizing training workshops on gene literature retrieval, profiling, and visualization for early career researchers

**DOI:** 10.12688/f1000research.36395.2

**Published:** 2023-05-11

**Authors:** Fatima Al Ali, Alexandra K Marr, Zohreh Tatari-Calderone, Mohamed Alfaki, Mohammed Toufiq, Jessica Roelands, Basirudeen Syed Ahamed Kabeer, Davide Bedognetti, Nico Marr, Mathieu Garand, Darawan Rinchai, Damien Chaussabel

**Affiliations:** 1 Research Branch, Sidra Medicine, Doha, Qatar; 2 College of Health and Life Sciences, Hamad Bin Khalifa University, Doha, Qatar; 3 Department of Internal Medicine and Medical Specialties, University of Genoa, Genoa, 16126, Italy

**Keywords:** Literature profiling, Science education, Concept extraction, Data visualization

## Abstract

Early-career researchers must acquire the skills necessary to effectively search and extract information from biomedical literature. This ability is for instance crucial for evaluating the novelty of experimental results, and assessing potential publishing opportunities. Given the rapidly growing volume of publications in the field of biomedical research, new systematic approaches need to be devised and adopted for the retrieval and curation of literature relevant to a specific theme. In this context, we present a hands-on training curriculum aimed at retrieval, profiling, and visualization of literature associated with a given topic. The curriculum was implemented in a workshop in January 2021. Here we provide supporting material and step-by-step implementation guidelines with the ISG15 gene literature serving as an illustrative use case. Workshop participants can learn several skills, including: 1) building and troubleshoot PubMed queries in order to retrieve the literature associated with a gene of interest; 2) identifying key concepts relevant to given themes (such as cell types, diseases, and biological processes); 3) measuring the prevalence of these concepts in the gene literature; 4) extracting key information from relevant articles, and 5) developing a background section or summary on the basis of this information. Finally, trainees can learn to consolidate the structured information captured through this process for presentation via an interactive web application.

## Introduction

Peer-reviewed publications constitute the body of knowledge upon which biomedical research is based. This resource is essential for the generation of novel hypotheses and the design of studies, experiments, and trials and is thus key to the discovery process itself. However, the volume of literature on any given research topic has grown dramatically in recent years, making it increasingly challenging for a single person to manually survey the relevant literature in its entirety, and consequently to acquire the foundational knowledge needed for discovery research. Hence, developing a solid foundation of skills for literature retrieval and profiling is of critical importance for early career biomedical scientists. These competencies will, for instance, be needed: 1) to acquire a sound knowledge base through developing the ability to compile and summarize large volumes of literature. 2) to develop data interpretation skills as well as become able to assess novelty and potential impact of a given finding; and 3) to develop scientific writing skills and become able to write background material on specific topics.

Publicly available omics data provides ideal material for training new generations of biomedical researchers. ^1– 3^ One of the “collective omics data” training modules that we have developed follows a reductionist analysis and interpretation workflow using publicly available transcriptome data as the source material. ^4^ The first activities that form part of this training module involve literature retrieval and profiling for a given candidate gene.

In this article, we describe a detailed stepwise approach that we have developed running literature profiling training workshops - literature profiling here meaning information extraction from titles and determination of keyword frequencies in titles and abstracts. The training program that we have devised takes advantage of publicly available omics data. ^4^ The training module presented here focuses on retrieval of gene-centric literature. Supporting material, such as sets of slides, templates, and a handout, is also provided along with an illustrative use case. Notably, although the training activity that is presented focuses on gene-centric literature retrieval and profiling, the skills and approaches can be adapted to any other application (e.g., focusing on disease-centric, or pathway/process-centric literature).

## Methods

The hands-on training exercise described here is suitable for implementation as part of a broader course on research methodology, or as a stand-alone workshop. The training is appropriate for undergraduate, graduate and post-doctoral trainees and no prior bioinformatics experience is required in order to participate. The time commitment depends on the level of experience of the attendees, the overall organization of the workflow and the volume of literature associated with the candidate gene(s) selected. For instance, for a gene with about 1,000 associated articles, five participants could work together on this same gene, each focusing on a different theme. Generally, the training could be covered in one introductory session (40 min), and three two-hour hands-on sessions. The format and content of these sessions are described in more detail below. These would cover:

• Introductory session
• Step 1 – Retrieving the relevant literature
• Step 2 – Extracting concepts
• Step 3 – Generating literature profiles
• Step 4 – Developing interactive data visualizations
• Step 5 – Writing a narrative

In addition to covering workshop pre-requisites and time commitment, announcements for such workshops can also list the sets of skills trainees are expected to develop including:

• Literature retrieval (development of advanced PubMed queries).
• Literature profiling (information extraction, determination of keyword frequencies).
• Data visualization (structuring and presenting information in an interactive format).
• Extraction of biomedical information (capturing information in a structured format).

Trainees can be asked to perform tasks between each of the sessions. Alternative formats are possible depending on the needs of the participants and a range of biomedical themes (e.g., diseases, pathways, or cell types) could be selected as the focus of the literature workshop. The endpoint for such workshops may include interactive literature profiling representations generated as part of the hands-on training activities and/or a peer-reviewed publication that, for instance, could build on such a resource.

### Introductory session

The introductory session is designed to provide participants with an understanding of basic concepts and present an outline of the training curriculum. In addition, this session presents the overall rationale and teaching objectives of the training program. The introduction also defines the endpoint of the workshop activity as the development of a web resource that permits the visualization and exploration of structured literature profiles for a gene of interest (described in detail below, taking *ISG15* as an illustrative case). This is followed by an overview of each of the steps that will be undertaken during the hands-on session. These are outlined above and described in more details below. It also provides participants with an opportunity to ask questions before the start of the hands-on sessions. Ideally, the presentation should last no longer than 40 minutes and an illustrative case could serve to support the presenter’s narrative. A generic presentation is provided ( **Extended data File 1)**. ^5^

### Step 1: Retrieving the relevant literature

As a first step, all the literature that is relevant to a given gene of interest must be identified. This forms the body of literature that will be subjected to literature profiling in subsequent steps. Most researchers will already be familiar with PubMed, the search engine hosted by the US National Center for Biotechnology Information (NCBI) ( https://pubmed.ncbi.nlm.nih.gov/). While this tool is straightforward to use, developing queries that will permit the comprehensive retrieval of literature associated with a given theme can be more complicated.

It is important to design a PubMed query that will permit the retrieval of all the literature that is available for the gene of interest. Challenges when retrieving literature associated with a given gene include capturing all aliases and variations that may exist in addition to the official gene names and symbols. For instance, 14 aliases were used to develop a query for the gene officially known as “ *ISG15* Ubiquitin Like Modifier”. Failure to include all the aliases can lead to under-representation of the literature and erroneous judgments that may have major consequences (e.g., when gaging the novelty of a given finding and its suitability for publication). The use of synonyms among gene names and aliases can also result in high proportions of false-positive results, particularly when aliases are common language terms (e.g., *CAMEL* genes [official symbol: *CTAG2*] or *WARS* genes [official symbol: *WARS1*]). If the literature retrieved in this step misses relevant records for the candidate gene (= false negatives), the resulting literature profiles will be incomplete. If the literature retrieved contains records that are irrelevant (= false positives), the resulting literature profiles will be inaccurate. For these reasons, it is important to optimize PubMed queries used for gene literature retrieval. Notably, the same principle applies when generating PubMed queries for any given topic. For instance, when querying PubMed to identify literature associated with inflammation, terms such as “inflammatory” or “inflamed” is also captured.

The endpoint of this step is the development of an optimized PubMed query for a given gene. Any criterion can be used for the selection of a candidate gene to be assigned to a trainee or group of trainees. However, a significant body of literature is associated with the gene in question. For the purposes of illustration in this article, we have selected *ISG15.*

Practical activities for this step:

- The official gene name, symbol and all aliases are retrieved from the GeneCard website (e.g., for *ISG15*: https://www.genecards.org/cgi-bin/carddisp.pl?gene=ISG15). Although gene names may also be retrieved from different sources, the advantage of using GeneCards is that information has already been compiled from reference databases such as NCBI Entrez Genes, Uniprot or genenames.org.
- PubMed queries are built using the official gene name, official symbol, and all aliases for the gene of interest as search terms, and by using the appropriate Boolean operators (AND, OR, NOT), field restriction tags, and suitable syntax. For instance, in a query using multiple search terms, the Boolean operator OR must be capitalized in between each keyword. The field restriction tag [tw] can be used after each term to search “text words”, included, for instance, in title, abstract, MeSH terms and subheadings, publication types, and substance names. Or alternatively the more restrictive [tiab], can be used in order to limit the search to titles and abstracts only. A query using [tw] for multiple search terms could for example be noted as follows: ISG15 [tw] OR IFI-15K [tw] OR IFI15 [tw]. In addition, the field restriction tag [pt], can be used to restrict/exclude the search of a particular publication type (e.g., NOT review [pt], would exclude review articles from the search results). Quotation marks are employed when compound words appear as an exact phrase in the search (e.g., “ *ISG15* Ubiquitin Like Modifier” [tw]). Notably, Pubmed’s advanced search builder can also be used to design such complex queries ( https://pubmed.ncbi.nlm.nih.gov/advanced/) And for reference, a wide range of training material can also be found on the NCBI website:
https://pubmed.ncbi.nlm.nih.gov/help/
https://learn.nlm.nih.gov/documentation/training-packets/T0042010P/
- The PubMed query is run, and quality checks are performed among the publications that were returned. This is to identify search terms that may be too permissive and return false-positive results. For instance, short three-character acronyms tend to be more problematic, as these are terms that are otherwise used as part of a common language (e.g. CAMEL, WARS).
- If necessary, the query is optimized by addressing problematic terms. Removing the ambiguous or problematic term altogether may be a solution, but this could also lead to false-negative results (missing literature that is actually relevant to the gene of interest). As a compromise, the search term could be retained but optimized by adding a keyword that would restrict the search (e.g., provided below for HUCRP and UCRP). One may also find in some instances that the list of aliases known for a given gene is incomplete and needs to be amended (e.g., below).

*Illustrative case:*

*The optimized query (searching all words and numbers in the title, abstract, other abstract, MeSH terms, MeSH subheadings, publication types, substance names, personal name as subject, corporate author, secondary source, comment/correction notes, and other terms) for ISG15 is as follows:*

*ISG15 [tw] OR “ISG15 Ubiquitin Like Modifier” [tw] OR "Interferon-stimulated gene 15" [tw] OR "IFN-induced 15-kDa protein" [tw] OR IFI-15K [tw] OR “Ubiquitin Cross-Reactive Protein” [tw] OR "Ubiquitin-Like Protein ISG15" [tw] OR (HUCRP [tw] AND "Cross-Reactive Protein" [tw]) OR G1P2 [tw] OR IP17 [tw] OR (UCRP [tw] AND "Cross-Reactive Protein" [tw]) OR "Interferon-Induced 17-KDa/15-KDa Protein" [tw] OR "Interferon-Stimulated Protein, 15 Kda" [tw] OR "Interferon-Induced 17 Kda Protein" [tw] OR IFI15 [tw] OR IMD38 [tw] NOT review [pt].*

*This query returned 1,186 results (as of September 1 ^st^, 2020). Optimization included the addition of aliases for this gene that were not captured in the GeneCard database but identified by reviewing some of the results (e.g., notably, the search argument Interferon-stimulated gene 15" [tw], which alone retrieves over 230 entries in PubMed). The acronyms HUCRP or UCRP also proved a source of false-positive results (CPR standing for cross-reactive protein, rather than C-reactive Protein). This was rectified by adding the ‘AND “cross reactive protein [tw]”’ argument to each of the ambiguous acronym.*

### Step 2: Extracting concepts

A large body of literature can be associated with a given gene. It may be useful then to employ a systematic “cataloguing” or indexing approach. For this step, workshop participants may first define themes under which concepts, and keywords associated with these concepts, will be categorized (e.g., themes could be ‘Human diseases and pathogens’, ‘Tissues’, ‘Cell types’, or ‘Cellular processes’). Participants would in turn scan titles of articles associated with *ISG15*, looking for concepts linked to the theme of interest. For example, under ‘Human diseases and pathogens’, a concept could be ‘liver cancer’ and associated keywords could be ‘liver cancer’, ‘hepatic carcinoma’, ‘hepatocellular carcinoma’, ‘HCC’, or ‘liver carcinoma’. The output for this activity would be lists of concepts and keywords associated with the gene literature organized under different themes. It is the prevalence of these concepts that will be measured in the subsequent step (generation of literature profiles).

Notably, this process could be repeated for other themes (or different themes could be assigned to each of the participants). If the literature is extensive and time is limited, it would also be possible to divide the literature into subsets (e.g., by batches of 100 articles, with participants assigned different batches to work on for the same theme). If the literature is sparse, all available articles for the gene may be used rather than just focusing on those articles including search terms in titles (i.e., using [tw], instead of [ti] in Step 2a). However, it may be generally preferable for a workshop to be based on selected genes with a relatively abundant literature (e.g., >100 articles returned when restricting the search to titles).

Practical activities for this step:

- A subset of the literature is retrieved, restricting the search to titles (using the optimized query from Step 1, and substituting the field restricting argument [ti] for [tw]).
- A given theme is assigned to, or selected by, participants (e.g., ‘Human diseases and pathogens’, ‘Tissues’, ‘Cell types’, ‘Biomolecules’, ‘Pathways’, ‘Biological processes’).
- Concepts relevant to the theme in question are identified in the titles of articles retrieved by the query designed in a) (e.g., liver cancer, HIV).
- The concepts and associated keywords (e.g., ‘hepatocellular carcinoma’, ‘liver carcinoma’, ‘hepatic cancer’, for the concept ‘liver cancer’ or ‘virus’, ‘viral’, ‘human immunodeficiency virus’ for the concept HIV) are recorded in a spreadsheet (example and templates are available in **Extended data File 2**). ^6^

*Illustrative case:*

*The subset of the ISG15 literature in which the official gene name, symbol or aliases are present in titles is retrieved. The query below is adapted to retrieve only the literature for which gene name and aliases are present in titles (using [ti]).*

*ISG15 [ti] OR “ISG15 Ubiquitin Like Modifier” [ti] OR "Interferon-stimulated gene 15" [ti] OR "IFN-induced 15-kDa protein" [ti] OR IFI-15K [ti] OR “Ubiquitin Cross-Reactive Protein” [ti] OR "Ubiquitin-Like Protein ISG15" [ti] OR (HUCRP [ti] AND "Cross-Reactive Protein" [tw]) OR G1P2 [ti] OR IP17 [ti] OR (UCRP [ti] AND "Cross-Reactive Protein" [tw]) OR "Interferon-Induced 17-KDa/15-KDa Protein" [ti] OR "Interferon-Stimulated Protein, 15 Kda" [ti] OR "Interferon-Induced 17 Kda Protein" [ti] OR IFI15 [ti] OR IMD38 [ti] NOT review [pt]. This query returned 312 results (as of September 1 ^st^, 2020).*

*Titles are then parsed from the ISG15 literature for concepts corresponding to the theme: “human diseases and pathogens”. The concepts and associated keywords retrieved for the “human diseases and pathogens” category are listed in **Extended data File 2** ^6^ (Excel File: Extraction of concepts Tab).*

### Step 3: Generating literature profiles

Determining the relative prevalence of concepts among the literature associated with a given gene can be useful. This is the case when assessing the novelty of a finding, for instance (e.g., change in transcript or protein abundance associated with pathogenesis). Moreover, such an exercise and the information being derived would also be useful in other instances when writing general background/summary about the gene for a report or a manuscript.

With the two previous steps completed, determining the prevalence of concepts in the literature associated with a given gene can be achieved quite simply. For this, the literature query developed in Step 1 is modified to narrow the search and retrieve literature associated with the concepts identified in Step 2. The endpoint for this activity is a table showing the frequency of articles for these concepts in the literature associated with a given gene (e.g., **Extended data File 2** ^6^ [Excel File: Literature Profiling Tab]).

**Figure 1.**
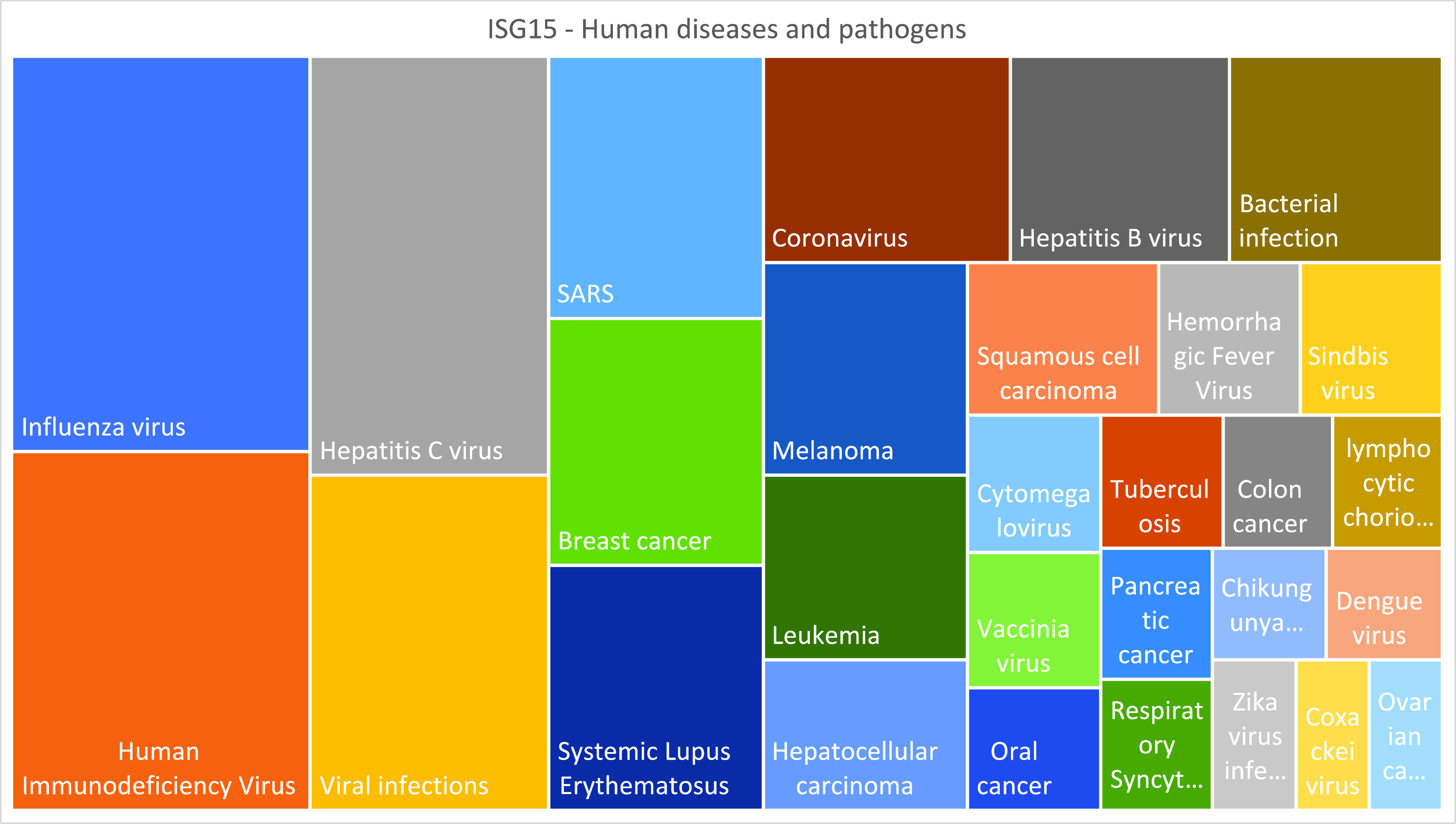
Visualizing *ISG15* literature profiles. Treemap representation of the relative prevalence of concepts associated with the “human diseases and pathogens” theme among the ISG15 literature.

Notes: Participants may also be encouraged to explore different types of visual representations for this type of data. Treemaps or word clouds can for instance show relative prevalence, while other types of graphs, such as 2D bubble graphs, may also show article frequencies (e.g., Figure 1). Another exercise could involve visualization of changes in the abundance of the literature associated with the selected concepts over the years. For this purpose, queries can be amended to add a range of publication dates with the field restriction tag [dp] (e.g., adding AND 2000:2010 [dp] to the query).

Practical activities for this step:

- The literature query from Step 1 is employed (using the field search restriction [tw]).
- The Boolean operator AND is added, followed by search terms corresponding to keywords related to one of the concepts identified in Step 2 (e.g., “Liver cancer”, “Liver carcinoma”, “Hepatic carcinoma”).
- Quotation marks, field search restriction and the Boolean OR are added, so that the notation would read as follows: … AND (“Liver cancer” [tw] OR “Liver carcinoma” [tw] OR “Hepatic carcinoma” [tw]).
- The query thus constructed is run, and the number of articles retrieved recorded (for instance in a spreadsheet: **Extended data File 2**). ^6^ Steps b through d are repeated for the rest of the concepts identified in Step 2.
- Visual representations of the literature profiles are generated.

*Illustrative case:*

*The prevalence of concepts among the ISG15 literature associated with the theme “Human diseases and pathogens” is given in **Extended data File 2** ^6^ (Excel File: Literature Profiling Tab) and represented visually in Figure 1 (also in **Extended data File 2** ^6^ [Excel File: Generating plots Tab]).*

### Step 4: Developing interactive data visualizations

Valuable insights can be gained from visual representation of information. And this could be further facilitated when the information underlying these visual representations can be accessed interactively by the end user.

**Figure 2.**
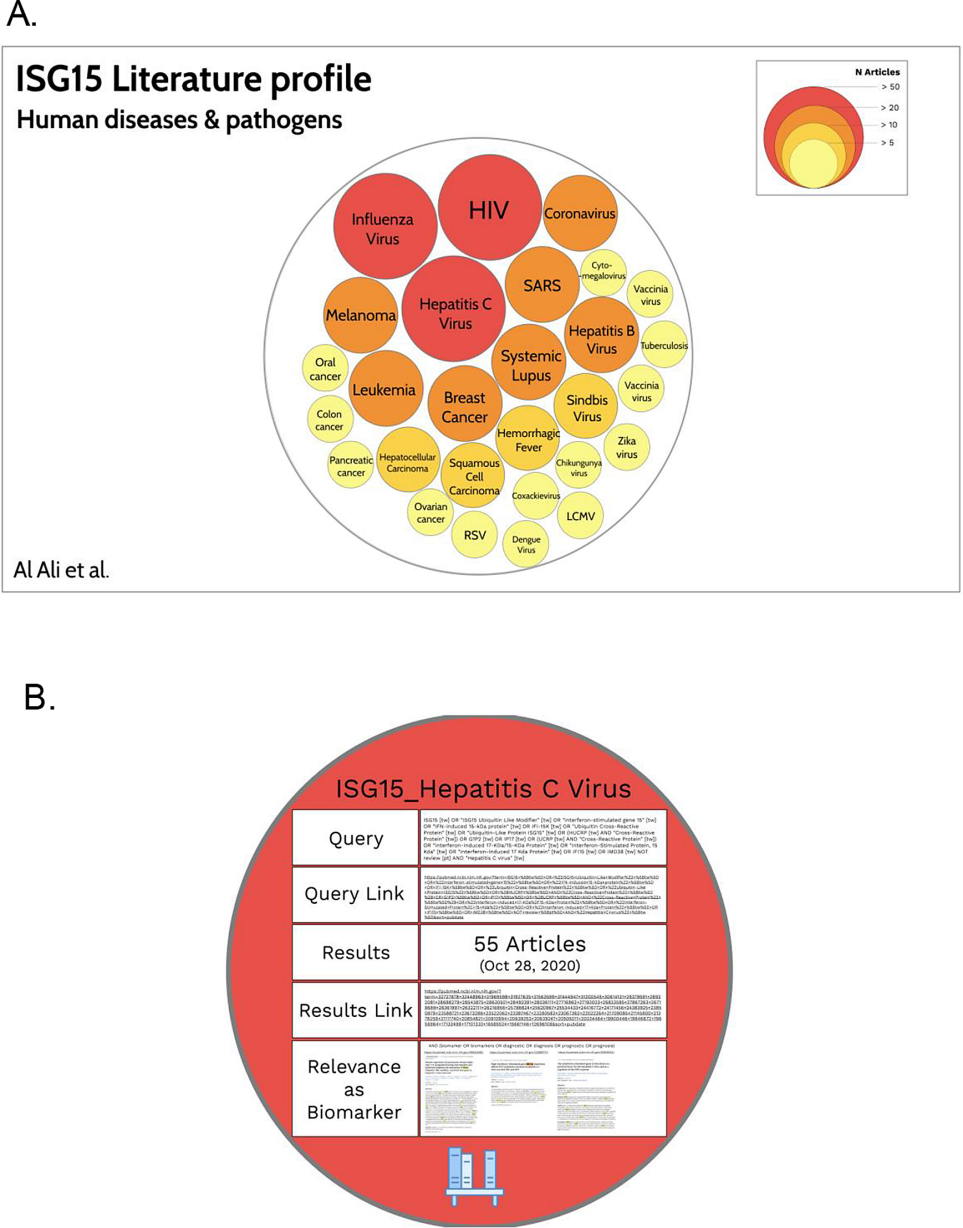
*ISG15* Human diseases and pathogens circle packing chart. A. At the highest level, the representation permits visualization of the relative abundance of concepts associated with the “human diseases and pathogens” theme for the *ISG15* gene. The color and size of the circles is a representation of the number of articles retrieved for a given concept (as indicated in the figure key). **B.** Zooming into each of the circles permits access to another level of information, such as links to PubMed results as well as screen captures of articles relevant to a specific topic (e.g., the relevance of *ISG15* as a biomarker). An interactive version of this presentation can be accessed via: *https://prezi.com/view/zCedrcYaAEUAON1VeEUi/*

To produce such interactive visual representations in the context of training workshops, we recommend using the Prezi web application ( https://prezi.com). This tool has been developed to create presentations in which it is possible to zoom in and out between levels of information. This gives users the opportunity to visualize the prevalence of concepts while at the same time allowing them to “drill down” into each individual concept in order to access relevant underlying information. The endpoint for this activity is the production of a “hierarchical circle packing chart” representing the relative abundance of concepts in the literature for a given gene and theme ( Figure 2A, https://prezi.com/view/zCedrcYaAEUAON1VeEUi). Such a resource gives users access to underlying reference information for each of the concepts ( Figure 2B) and can be made available publicly.

For the practical activity designed for this step, participants will need to register with Prezi and create an account to access and edit the interactive presentations. They can do so free of charge by selecting the basic account at: https://prezi.com/pricing/basic/. Ideally, this is completed ahead of the training session. If instructors have access to a paid subscription, they can create an unlimited number of presentations and invite individual participants to collaborate with editing rights. Otherwise, the participants can create their own presentation and invite the instructors as collaborators. They would also need to copy and paste material from a master template in their own Prezi account. Thus, this alternative solution would be workable, but probably not ideal.

Practical activities for this step:

- Participants are given access to a Prezi presentation that contains a template and illustrative example (e.g. https://prezi.com/view/u65ZqHn9ZBJx1VKHqfZA/). As indicated above, one such presentation could be created and made available for each participant. It could serve as their own “Sandbox”, to familiarize themselves with the application and use as a starting point to develop an interactive resource. Multiple users can also work simultaneously on the same shared presentation. It would thus also be possible to create one presentation for a group of participants to work on cooperatively.
- Starting with the template and following the illustrative example, participants can build a circle packing chart for concepts identified in Step 3.
- The size and color of the circles is determined by the frequency of articles in the gene-associated literature related to a given concept (as shown in Figure 2A).
- The circles can be arranged manually to create a visually attractive representation.
- Underlying information is then added to each of the circles and accessed by zooming in to different levels ( Figure 2B). This information could include, for instance:
  • The gene symbol and concept;
  • The query link;
  • Number of articles retrieved on a specified date;
  • Result link to those articles;
  • Screen capture and links pointing to articles relevant to a specific topic (see Step 5 below).

*Illustrative case:*

*The circle packing chart representation for ISG15, focusing on the “Human diseases and pathogens” topic is shown in* *Figure 2* *. The Prezi presentation can be accessed for interactive exploration via:* https://prezi.com/view/zCedrcYaAEUAON1VeEUi/

A screen recording demonstrating how to add underlying information to a Prezi circle packing chart can be accessed via: https://soapbox.wistia.com/videos/2tmC1VeQyr

### Step 5: Writing a narrative

Writing material for a report or manuscript can be one of the motivations for profiling the literature associated with a given topic. To become successful in their future academic endeavors early career scientists need to become proficient independent writers. The last hands-on activity of the workshop consists in capturing key information from published work and using it as a basis for writing up a narrative about the gene of interest.

A specific topic would be selected as the focus of the activity to limit the amount of literature that would need to be covered. One such topic could, for example, be the relevance of a gene (in this case *ISG15*) as a biomarker for diagnostic applications within the overall theme of “Human diseases and pathogens” defined in Step 2. Participants could then focus on the most prevalent disease (e.g. in the case of *ISG15* literature, three diseases have >50 articles and ten diseases have >20 articles). Literature queries corresponding to the diseases that have been selected would then be modified to retrieve only those most likely to contain information about *ISG15* and its relevance as a biomarker.

Practical activities for this step:

- Concepts most prevalent in the gene-associated literature for the theme of interest are selected (e.g. concept = Hepatitis C infection, within the theme = “Human diseases and pathogens”).
- The query developed to retrieve literature for a given concept is modified to restrict the search in order to retrieve articles relevant to a given topic (e.g. relevance of the gene as a biomarker). The Boolean “AND” is appended at the end of the query along with relevant keywords in parenthesis separated by the Boolean “OR” (e.g. “AND (biomarker OR biomarkers OR diagnostic OR diagnosis OR prognostic OR prognosis)”.
- Articles are reviewed and those deemed relevant to the topic are selected (e.g., those in which changes in abundance of the gene product in clinical specimens are reported).
- Relevant information is captured from the abstract and/or full text and recorded in a spreadsheet (e.g. analyte name, species, data generation methods, comparator groups, etc.). In some cases, the full text of articles may not be accessible (e.g., paywall), and the abstract has only incomplete information. This can lead to the absence of important information in the “capture” spreadsheet, rendering the findings unusable. Participants can then be reminded of the importance of using best practices when reporting findings (e.g., mentioning the comparator group, species or the specific factor being measured – being protein or RNA). It may also be an opportunity to discuss the merits of publication in open access journals.
- The information captured in the spreadsheet will serve as a synopsis that participants will be able to rely upon for developing a written narrative, which could be used in an introduction or review article.

*Illustrative case:*

*Gene of interest: ISG15*

*Theme: “Human diseases and pathogens”.*

*Topic: Biomarker/diagnostic relevance.*

*Focusing on ISG15 and “human diseases and pathogens” as a theme, with biomarker/diagnostic/prognostic as a topic, the main diseases identified in step 3 were selected (>20 articles; Figure 2A, red circles and orange circles). Starting with the query developed for retrieving ISG15 literature relevant to Hepatitis C virus, the search is restricted by adding the expression “AND (biomarker OR biomarkers OR diagnostic OR diagnosis OR prognostic OR prognosis)”. Three articles were returned. From each article, relevant information available in the abstract, and in the full text where possible/necessary, is recorded in a spreadsheet ( **Extended data File 3**). ^7^ The information in question relates specifically to changes in abundance of ISG15 measured in clinical specimens (since it is directly relevant to the topic that was selected).*

*Narrative, based on the information captured in the spreadsheet:*

*“Interferon-stimulated gene 15 (ISG15) is a member of the ubiquitin family, which includes ubiquitin and ubiquitin-like modifiers (Ubls). Ubiquitin and Ubls are involved in the regulation of a variety of cellular activities, including protein stability, intracellular trafficking, cell cycle control and immune modulation. ISG15 has been implicated as a biomarker with diagnostic relevance for a number of human disorders, including cancer (melanoma and breast cancer) and autoimmune diseases (SLE), as well as infection with pathogens such as HBC, HCV and HIV.*

*The expression of ISG15 has been implicated in a wide range of human cancers, although the roles of ISG15 in tumorigenesis and responses to anticancer treatments remain largely unknown. In patients with breast cancer, ISG15 is overexpressed at both the mRNA and protein levels in mammary tumor tissue compared to that in normal mammary tissue. ^8, 9^ Increased ISG15 protein expression has also been detected in human melanoma cell lines. ^10^ These findings indicate the potential of ISG15 as a tumor biomarker. ISG15 protein expression is upregulated in immune cell markers from patients with invasive breast cancer ^11^ as well as dendritic cells from melanoma patients. ^10^ ISG15 is also highly expressed in breast cancer patients undergoing immunotherapy. ^12^ Short-hairpin RNA-mediated silencing of ISG15 expression has been shown to inhibits breast tumor growth and increase NK cell infiltration a nude mouse model of tumorigenesis. ^13^ Furthermore, shRNA-mediated knockdown of ISG15 increased the resistance of human tumor cells to CPT. ^14^ These findings indicate that ISG15 is also a candidate biomarker of the responsiveness to immunotherapy among patients with cancer.*

*ISG15 plays a key role in the host antiviral response. As such, ISG15 is implicated as a diagnostic biomarker of viral infection. Studies in patients with HCC have shown high levels of ISG15 protein in human HBV-related HCC tissues compared to the levels detected in non-tumor tissues. ^15^ Furthermore, upregulated expression of ISG15 was observed at both the mRNA and protein levels in liver tissue samples from patients with HCV infection compared to the levels detected in uninfected controls. ^16^ Similarly, the abundance of ISG15 transcripts was found to be increased in human PBMCs and liver cells in patients with HCV infection who were unresponsive to IFN treatment compared to the levels in corresponding IFN-responsive patients. ^17^ In addition, high levels of ISG15 in the liver of patients with HCV infection were associated with an unfavourable HCV genotype 1, a high hepatic HCV load and a low antiviral response to IFN compared to patients who did not present such characteristics. ^16^ These findings indicate the potential of ISG15 as a biomarker of IFN treatment response in patients with HCV infection.*

*Increased ISG15 expression has also been reported in mouse models related to HIV infection. ISG15 is upregulated in transgenic mice overexpressing the HIV gp120 protein. ^18^ Moreover, in mouse models of acute and chronic neuronal injuries with HIV infection, ISG15 protein in the brain was increased compared to the levels detected in mice with global ischemia and traumatic brain injuries. ^18^ The importance of ISG15 as a biomarker of HIV infection and treatment response has also been revealed in human studies. Compared to healthy donors, TaqMan assays revealed high ISG15 expression (mRNA and protein) as well as an increased frequency of ISG15-SNPs in human PBMC from untreated patients with HIV infection. ^19^ Moreover, ISG15 expression decreased in human PBMC in patients with HIV infection after long-term antiretroviral therapy. ^19^*

*The key importance of ISG15 in the human immune system is also reflected in its role in autoimmune diseases, such as SLE. High levels of ISG15 transcripts were detected in human whole blood cell samples from 28 patients newly diagnosed with SLE compared to with 10 patients with undifferentiated connective tissue disease, and 22 healthy volunteers, ^20^ indicating the potential of ISG15 as a specific biomarker of SLE. Moreover, the upregulation of ISG15 in human plasmablasts/plasma cells from patients with active SLE indicate that ISG15 can be used as a marker of disease activation in patients in remission. ^21^*

*Thus, an increasing body of evidence supports a role of ISG15 as a biomarker with diagnostic relevance for a number of human disorders.”*

## Implementation

As proof of principle, a workshop was implemented using the step-by-step guide and supporting information provided with this paper. It was led by FAA and AKM, who participated in the development of the training curriculum, but had no prior experience running such training activities. The workshop took place on January 26, 2021. In total, 29 graduate students from Hamad Bin Khalifa University took part. It was offered as one three-hour class as part of an “Introduction to Data Science” course. Due to Covid-19 restrictions the workshop was run remotely using Webex. As no information was collected from workshop participants (i.e., no surveys, or questionnaires), the activity was not considered to constitute human research and therefore no ethical approval was required. The generic introductory presentation in **Extended data File 1** ^5^ was adapted to provide more specific context, and notably explain how, *CCR1*, the target gene for this workshop was selected (for illustrative purposes this introductory presentation is also made available here: **Extended data File 4**). ^22^ FAA and AKM, the two co-instructors, also prepared a handout to guide participants through the different steps ( **Extended data File 5)**, ^23^ specifically for the *CCR1* use case. Several days prior to the workshop, participants had been asked to create a Prezi “Basic” account and a template was created and shared with each one of them ahead of time. Following the introductory presentation (10 minutes) participants carried out hands-on activities following each one of the tasks described in the handout, with each step corresponding to one working session. Given time constraints, material prepared ahead of time by FAA and AKM was provided to the participants at the end of each working session so that they could move on to the next. Ideally sessions should span multiple days in order for participants to complete the assignments. However, the more intensive schedule implemented here had the advantage of introducing literature profiling concepts and approaches to a large group of trainees in a time-effective manner. The opportunity to continue work later on, on their own time being also available to them.

## Conclusions

Effectively harnessing biomedical literature is one of the most fundamental skills required by biomedical researchers. Thus, early career researchers need to develop the ability to read the scientific literature at different levels: from “scanning” a large number of articles to reading the full text version for a more in depth understanding. Given the current rate at which articles are published, developing more systematic approaches to literature profiling based on defined principles may prove especially beneficial to early-career biomedical researchers.

Here, we present a training workflow as well as supporting material that may be re-used/adapted for the organization of ‘Gene literature retrieval, profiling and visualization’ training workshops. Hands-on activities range from literature retrieval and optimization of PubMed queries, to the development of interactive resources and authoring original material focusing on a specific topic.

A number of the steps described could easily be automated. For instance, the concatenation of official gene names and aliases for retrieving gene-specific literature using PubMed (Step 1). Indeed, such tools are under development by our group and will be made available in the future. These tools would not only save time, but also minimize user error. Nevertheless, some of the steps, such as optimization of search queries or extraction of information from abstracts will always require some critical evaluation and decision making that cannot be automated. Refraining from the use of such automated tools in a training workshop may also be a deliberate choice on the part of the instructor if the emphasis is initially on the development of competency in completing the steps involved throughout the process.

Here, we focused on gene-associated literature, simply because the training curriculum that is currently under development is based on the reuse of publicly available gene expression data. A similar approach could be employed to profile the literature associated with any given disease, pathway, molecular process, or drug, for instance.

We aim to further develop an illustrative case that would lead to the publication of a review of the *ISG15* literature employing the literature profiling and visualization approaches described herein. We also plan in the future to develop and make available guides and supporting material for the implementation of hands-on training workshops focusing on other topics covered by our Collective Omics Data (COD) training curriculum, which includes three modules: COD 1 focuses on retrieving, visualizing and interpretating gene-specific transcriptional profiles ^24– 26^; COD2 focuses on constituting and curating of themed public dataset collections ^27– 35^; COD3 focuses on global analysis of large-scale omics data. ^36– 38^ These hands-on training activities would build in part on the skills developed through the implementation of the literature profiling workshop that we have presented here. Progress to date towards this goal includes the publication of a study guide for COD1, ^39^ along with a use case that examines the relevance of CEACAM6 as a blood transcriptional marker. ^40^

## Data availability

### Underlying data

No newly generated data are associated with this article. Information was retrieved via the literature search engine PubMed ( https://pubmed.ncbi.nlm.nih.gov/).

### Extended data

This project contains the following extended data:

Figshare: Literature profiling workshop, introduction session slides (S File 1), https://doi.org/10.6084/m9.figshare.13669070.v2. ^5^

Figshare: Literature profiling workshop: steps 1-3 (S File 2), https://doi.org/10.6084/m9.figshare.14160329.v3. ^6^

Figshare: Literature Profiling Workshop: Step 5 (S File 3), https://doi.org/10.6084/m9.figshare.14161484.v1. ^7^

Figshare: Literature profiling workshop: HBKU handout (S File 4), https://doi.org/10.6084/m9.figshare.14166395.v1. ^22^

Figshare: Literature profiling workshop: HBKU intro presentation (S File 5), https://doi.org/10.6084/m9.figshare.14166500.v1. ^23^

Data are available under the terms of the Creative Commons Attribution 4.0 International license (CC BY 4.0).
