# Supplementary material for: Organizing training workshops on gene literature retrieval, profiling, and visualization for early career researchers
Source: F1000Res. 2023 May 11;10:275. Originally published 2021 Apr 6. [Version 2] doi: 10.12688/f1000research.36395.2 (PMC10336363; doi:10.12688/f1000research.36395.2)
Supplement: Peer review history

# Peer review: Reviewer response for version 2

**DOI:** 10.5256/f1000research.145671.r173013

**Published:** 2023-05-25

**Authors:** Valerie Matarese

**Affiliations:** 1 Authors' editor and editorial consultant, Self-employed, Vidor TV, Italy

I have read the revised manuscript and am pleased to see that the authors acted on most of my comments. As a result, the manuscript has improved on many levels. The inclusion of this manuscript in the literature on scientific writing workshops will be useful to the scientific community, as it illustrates some unique approaches and viewpoints. I therefore give my approval to the manuscript and look forward to seeing it indexed in bibliographic databases.

Is the rationale for developing the new method (or application) clearly explained?

Partly

Is the description of the method technically sound?

Yes

Are the conclusions about the method and its performance adequately supported by the findings presented in the article?

Partly

If any results are presented, are all the source data underlying the results available to ensure full reproducibility?

Yes

Are sufficient details provided to allow replication of the method development and its use by others?

Yes

Reviewer Expertise:

I am a former biomedical researcher with 25 years' experience as a biomedical editor in different capacities and 15 years' experience as instructor of research writing at the PhD level. I am also a scholar of academic editing.

I confirm that I have read this submission and believe that I have an appropriate level of expertise to confirm that it is of an acceptable scientific standard.

# Peer review: Reviewer response for version 1

**DOI:** 10.5256/f1000research.39457.r96508

**Published:** 2021-10-20

**Authors:** Valerie Matarese

**Affiliations:** 1 Authors' editor and editorial consultant, Self-employed, Vidor TV, Italy

**Overview**

 This method article describes a short workshop (3 hours) with five stated teaching objectives: bibliographic research in PubMed on gene-related topics (“retrieval”); extraction of relevant keywords (“concepts”) from article titles; “profiling” (counting the number of articles per concept); using the data and online tools to create interactive graphics (“visualization”); and “writing a narrative review” using a semi-automated method in a spreadsheet. I welcome a workshop description for teaching bibliographic research, and I am sure that workshop trainees will enjoy learning to make treemaps and circle packing charts. However, I have substantial reservations about the last objective and thus, as peer reviewer, I cannot endorse the method as a whole.

**Regarding F1000Research questions**

 The rationale for the workshop is clearly given, but no valid justification is given for using a spreadsheet to write a narrative review (fifth objective) for the Introduction section of a research article or for a review article. The conclusions about the method and its performance are supported in part by the findings presented in the article, but the generated nonsense text is clear evidence that the proposed method for automated text generation should not be published.

**Major comment**

 The fifth objective, “Writing a narrative”, is associated with two skills that workshop trainees can expect to learn: “Assimilation of biomedical knowledge (synthesis and presentation)” and “Scientific publication (optional)”. However, as described in the manuscript, the workshop is unable to fulfill these promises at all. In this fifth part of the workshop, participants are instructed to read the “abstract and/or full text and record in a spreadsheet” a series of data. They then use the spreadsheet’s concatenate function to generate “standard sentences” and collate these into a “cohesive paragraph or section”. Finally, the text is “polished” by the students, an instructor or a “professional scientific copyeditor”. There are enormous problems with all of this.

 First and foremost, the method is proposing the automated generation of text for publication based primarily on abstracts (“full text where possible/necessary”) and without critical appraisal. Only in the conclusions of the manuscript is there any minimal mention of this problem: “Nevertheless, … extraction of information *from abstracts* will always require *some* critical evaluation and decision making that cannot be automated” (emphasis added). I cannot endorse a method for the computer-assisted writing of research articles and reviews, as it is unscholarly, wasteful to the reviewers and (if published) readers who will have to sift through substantial nonsense text, and damaging to knowledge advancement.

 That this method can generate nonsense text is seen in the two pages of “Illustrative case”. Part A (page 11) shows the computer-generated “standard sentences” which are close to gibberish, for example:

*The abundance of ISG15 Proteins measured by immunohistochemistry is increased in vivo in human immune cell markers “in patients with” invasive BC patients with long-term follow-up compared to … … ………… … .. [33073304].*

 Part B (page 12) shows the “edited narrative” which “was prepared by a professional scientific copyeditor”. Given both the poor science and poor English grammar and style of this narrative, I doubt that a professional editor worked on it. Furthermore, the use of an editor to create meaningful text from the “standard sentences” in Part A would amount to ghost writing. Here is just one example from the supposedly edited text:

*Compared to healthy donors, TaqMan assays revealed high ISG15 expression (mRNA and protein) as well as an increased frequency of ISG15-SNPs in human PBMC from untreated patients with HIV infection.*

 TaqMan assays do not inform on protein levels. The frequency of a SNP cannot change unless there is gene duplication (what changes is the allele frequency). There is no need to use “human” to describe cells from HIV patients. There is a misplaced modifier before “TaqMan” and an unwanted hyphen.

 I therefore feel that, for a revised version of this manuscript to be approved, the fifth objective should be deleted along with its two associated skills (impossible to achieve in a short workshop). In addition, a paragraph should be added with comments on the need for early career researchers to learn to read the scientific literature at different levels (from scanning to critical appraisal *of the full text*) and to appreciate that scientific findings must be replicated and validated before they can be considered “true”.

**Minor comments**

 The title is a hard read, because there are six words between “Organizing” and what gets organized. I suggest: Organizing training workshops on gene literature …. for early career researchers”.

 The word “profiling” is used to the point of being meaningless. It is used six times in the 20-line Introduction, in the context of literature, omics and transcriptome. It should be deleted after “omics” and “transcriptome”, as these words are best used as nouns, not adjectives describing the vague “profiling”. Regarding “literature profiling”, this non-standard expression should be defined early in the text, not in the Methods section (bottom page 3). Here, profiling means “information extraction” from titles and “determination of keyword frequencies”. This definition should be given in the Introduction as soon as “literature profiling” is mentioned.

 Throughout the text: The use of conditional mood (“this session should present”, “the presentation should last” and so on) is unnecessary, burdens the text and slows reading. The simple present tense can be used in most cases. A method is not a recommendation (“should”) but a description (“is”).

 Throughout the text: The use of italics in the “illustrative cases” makes reading difficult, especially because of the extensive use of quotation marks and parentheses. Italics should be reserved for emphasis on small pieces of text. Here, instead, a block quote (indented text) would be better. Text boxes can also help.

 Page 2: “some independent preparation”. Does this mean independent study, exercises, getting organized technically?

 Page 2: Ref. 34 is cited right after Ref. 4. It should be renumbered 5. I confused it with Refs. 3,4 (missing a comma). Similarly Ref. 35 is cited too early, on page 6.

 Page 3, line 3: “on any given specific research topics” should be changed to “on any given research topic”. *Topic* should be singular. *Specific* is unneeded and redundant with “given topic”.

 Page 3: “for a gene with ± 1000 associated articles and five participants”. This phrase is too concise to have meaning, as a gene cannot have participants.

 Page 4, Step 1. No mention is made of NCBI’s Advanced Search Builder; MeSH terms; or truncation by asterisk. The manuscript should explain why these advanced features are not taught.

 Page 4, bottom: “The field restriction tag [tw] (or alternatively the more restrictive [tiab]) should be added after each term… to limit the search to titles and abstracts”. Actually, it is [tiab] that limits the search to titles and abstracts. The broad [tw] searches almost everything (see https://pubmed.ncbi.nlm.nih.gov/help/#tw).

 Page 5: “examples provided above”. Where? Vague cross-references like this make reading difficult.

 Page 6: “For instance, when assessing the novelty …”. This incomplete sentence is lacking a subject-verb pair. Similar problem on page 13, bottom.

 Page 6, bottom: “The Boolean argument AND is added”. AND is a Boolean operator, not argument.

 Page 6, bottom: ‘… AND (“Liver cancer”[tw] OR “Liver carcinoma”[tw] OR “Hepatic carcinoma”[tw], etc.)’. A query cannot contain “, etc.”. It should be removed, and a final period added to the sentence.

 Page 9, Step 5. This part is excessively wordy and suggests an uneasiness with the concepts. We read “is the measure by which productivity is measured”, “it can be at first important to be able to identify information that is worth capturing” and “A specific topic would be selected as the focus … to limit the amount of literature that would need to be covered”.

 Page 13: Webex. Not WebEx.

 Punctuation

- Comma after “Supporting material” in the third paragraph of Introduction. Comma after “This is a skill that is worth developing” (page 9).
- Replace ± with “about” (first paragraph of Methods).
- Hyphens: no hyphen after -ly adverbs, e.g. “early career researcher”. Use hyphens in “gene-associated literature”, “false-positive result” and “false-negative result” (but not in “false positives” or “false negatives”).
- There are many instances where the required space between words is missing, especially when a quotation mark is used, e.g. “For example, under’Human diseases and … associated keywords could be’liver cancer’,’hepatic carcinoma’, …” (page 5). The entire manuscript should be checked and spaces added where needed. A hint is that the curly quotation mark is in the wrong direction before the word when a space is missing.
- Double quotation marks should be used in all cases except when nesting is needed. Nesting should be used on page 5 where the sentence:
*This was rectified by adding the AND “cross-reactive protein [tw] argument to each of the ambiguous acronym. *
  should be changed to:
*This was rectified by adding the ‘AND “cross-reactive protein [tw]”’ argument to each of the ambiguous acronyms.*
  or more simply:
*This was rectified by adding ‘AND “cross-reactive protein [tw]”’ to each of the ambiguous acronyms.*

Is the rationale for developing the new method (or application) clearly explained?

Partly

Is the description of the method technically sound?

Yes

Are the conclusions about the method and its performance adequately supported by the findings presented in the article?

Partly

If any results are presented, are all the source data underlying the results available to ensure full reproducibility?

Yes

Are sufficient details provided to allow replication of the method development and its use by others?

Yes

Reviewer Expertise:

I am a former biomedical researcher with 25 years' experience as a biomedical editor in different capacities and 15 years' experience as instructor of research writing at the PhD level. I am also a scholar of academic editing.

I confirm that I have read this submission and believe that I have an appropriate level of expertise to state that I do not consider it to be of an acceptable scientific standard, for reasons outlined above.

# Response

**Published:** 2023-03-17

**Authors:** Damien Chaussabel

**Affiliations:** The Jackson Laboratory for Genomic Medicine, USA

**Overview**

**This method article describes a short workshop (3 hours) with five stated teaching objectives: bibliographic research in PubMed on gene-related topics (“retrieval”); extraction of relevant keywords (“concepts”) from article titles; “profiling” (counting the number of articles per concept); using the data and online tools to create interactive graphics (“visualization”); and “writing a narrative review” using a semi-automated method in a spreadsheet. I welcome a workshop description for teaching bibliographic research, and I am sure that workshop trainees will enjoy learning to make treemaps and circle packing charts. However, I have substantial reservations about the last objective and thus, as peer reviewer, I cannot endorse the method as a whole.**

 We appreciate the time spent reviewing this work and the constructive feedback given. The reason we are coming back to revising this article a little bit late is because we wanted first to complete the development of a more rounded training curriculum. As a result, a study guide and use case have recently been published in F1000Research ( https://f1000research.com/articles/11-994, https://f1000research.com/articles/11-1294). Literature profiling remains nonetheless a major component of this training curriculum and could be run as stand-alone training/workshop. Also, we also wanted to circle back to this manuscript at this time and address all outstanding comments and add references to the more recent work.

 We have addressed the main comment raised in the review by eliminating the step in the workflow that involved automatically generating text from the information provided by workshop participants. We did not intend for this step to cause controversy and were simply trying to practically train early-career scientists in countries where English literacy may not be as high as in more privileged countries. The question then becomes, how can these brilliant scientists overcome the language barrier and fulfil their potential? The approach we attempted, as an experiment, was only meant to be a first step towards trainees becoming independent writers, eventually able to produce literary work without artificial crutches. However, we admit that the approach may have been flawed or misguided (or both!), and we acknowledge your point. We are happy to remove this step from the manuscript.

**Regarding F1000Research questions**

**The rationale for the workshop is clearly given, but no valid justification is given for using a spreadsheet to write a narrative review (fifth objective) for the Introduction section of a research article or for a review article. The conclusions about the method and its performance are supported in part by the findings presented in the article, but the generated nonsense text is clear evidence that the proposed method for automated text generation should not be published.**

 Thanks for raising these concerns. We have now removed the steps that pertains to automated text generation (5e and 5f). We feel that capturing relevant information and recording it in a spreadsheet is still a worthwhile exercise. Indeed, participants can learn in the process what information they should be looking for in the work of others and make sure to report in their own (e.g. abstracts often fail to mention in what species the work is performed or if abundance of protein or transcripts is measured). We now write instead as step 5e:

 “The information captured in the spreadsheet will serve as a synopsis that participants will be able to rely upon for developing a written narrative, which could be used in an introduction or review article.”

 The spreadsheet has also been amended accordingly, removing the functions used to generate text. And the text that was automatically generated has been removed from the manuscript. We retained the written narrative, simply prefacing the paragraph with the following statement:

*“Narrative, based on the information captured in the spreadsheet:* “

**Major comment**

**The fifth objective, “Writing a narrative”, is associated with two skills that workshop trainees can expect to learn: “Assimilation of biomedical knowledge (synthesis and presentation)” and “Scientific publication (optional)”. However, as described in the manuscript, the workshop is unable to fulfill these promises at all. In this fifth part of the workshop, participants are instructed to read the “abstract and/or full text and record in a spreadsheet” a series of data. They then use the spreadsheet’s concatenate function to generate “standard sentences” and collate these into a “cohesive paragraph or section”. Finally, the text is “polished” by the students, an instructor or a “professional scientific copyeditor”. There are enormous problems with all of this.**

**First and foremost, the method is proposing the automated generation of text for publication based primarily on abstracts (“full text where possible/necessary”) and without critical appraisal. Only in the conclusions of the manuscript is there any minimal mention of this problem: “Nevertheless, … extraction of information *from abstracts* will always require *some* critical evaluation and decision making that cannot be automated” (emphasis added). I cannot endorse a method for the computer-assisted writing of research articles and reviews, as it is unscholarly, wasteful to the reviewers and (if published) readers who will have to sift through substantial nonsense text, and damaging to knowledge advancement.**

**That this method can generate nonsense text is seen in the two pages of “Illustrative case”. Part A (page 11) shows the computer-generated “standard sentences” which are close to gibberish, for example:**

***The abundance of ISG15 Proteins measured by immunohistochemistry is increased in vivo in human immune cell markers “in patients with” invasive BC patients with long-term follow-up compared to … … ………… … .. [33073304].***

**Part B (page 12) shows the “edited narrative” which “was prepared by a professional scientific copyeditor”. Given both the poor science and poor English grammar and style of this narrative, I doubt that a professional editor worked on it. Furthermore, the use of an editor to create meaningful text from the “standard sentences” in Part A would amount to ghost writing. Here is just one example from the supposedly edited text:**

***Compared to healthy donors, TaqMan assays revealed high ISG15 expression (mRNA and protein) as well as an increased frequency of ISG15-SNPs in human PBMC from untreated patients with HIV infection.***

**TaqMan assays do not inform on protein levels. The frequency of a SNP cannot change unless there is gene duplication (what changes is the allele frequency). There is no need to use “human” to describe cells from HIV patients. There is a misplaced modifier before “TaqMan” and an unwanted hyphen.**

**I therefore feel that, for a revised version of this manuscript to be approved, the fifth objective should be deleted along with its two associated skills (impossible to achieve in a short workshop). In addition, a paragraph should be added with comments on the need for early career researchers to learn to read the scientific literature at different levels (from scanning to critical appraisal *of the full text*) and to appreciate that scientific findings must be replicated and validated before they can be considered “true”.**

 We amended Step 5 as described above, removing altogether any notion of automated text generation, while keeping the step that consists in capturing and recording key information in a spreadsheet. If necessary, even this could be removed, but again as stated above we feel it is a worthwhile exercise.

 We amended our learning objectives, removing the two points mentioned in your critique and writing instead:

- Extraction of biomedical information (capturing information in a structured format).

We also added wording highlighting the need for participants to go beyond scanning titles and abstracts and read research papers:

 “Thus, early career researchers need to develop the ability to read the scientific literature at different levels: from “scanning” a large number of articles to reading the full text version for a more in depth understanding.”

 And for the record – since integrity is no small matter in science - we maintain that a professional and reputable science editor did work on this text, as we had stated earlier; even though as pointed out the service rendered was maybe not of the highest quality in this case (we have been otherwise been quite happy with them).

**Minor comments**

**The title is a hard read, because there are six words between “Organizing” and what gets organized. I suggest: Organizing training workshops on gene literature …. for early career researchers”.**

 Thank you for the helpful edits, which have been incorporated throughout the text and to the best of our abilities. We also took your advice and changed the title of the article to improve legibility.

**The word “profiling” is used to the point of being meaningless. It is used six times in the 20-line Introduction, in the context of literature, omics and transcriptome. It should be deleted after “omics” and “transcriptome”, as these words are best used as nouns, not adjectives describing the vague “profiling”. Regarding “literature profiling”, this non-standard expression should be defined early in the text, not in the Methods section (bottom page 3). Here, profiling means “information extraction” from titles and “determination of keyword frequencies”. This definition should be given in the Introduction as soon as “literature profiling” is mentioned.**

 We have deleted profiling when associated with omics data and defined literature profiling early on in the introduction.

**Throughout the text: The use of conditional mood (“this session should present”, “the presentation should last” and so on) is unnecessary, burdens the text and slows reading. The simple present tense can be used in most cases. A method is not a recommendation (“should”) but a description (“is”).**

 That makes sense and we have edited the text throughout.

**Throughout the text: The use of italics in the “illustrative cases” makes reading difficult, especially because of the extensive use of quotation marks and parentheses. Italics should be reserved for emphasis on small pieces of text. Here, instead, a block quote (indented text) would be better. Text boxes can also help.**

 Text boxes sound ideal. We will reach out to the editor to see if this is feasible.

**Page 2: “some independent preparation”. Does this mean independent study, exercises, getting organized technically?**

 We clarified this point by rewording this sentence, that now reads: “Trainees can be asked to perform tasks between each of the sessions.”

**Page 2: Ref. 34 is cited right after Ref. 4. It should be renumbered 5. I confused it with Refs. 3,4 (missing a comma). Similarly Ref. 35 is cited too early, on page 6.**

 Indeed, we will communicate this to the editor so it can be addressed in the formatted document.

**Page 3, line 3: “on any given specific research topics” should be changed to “on any given research topic”. *Topic* should be singular. *Specific* is unneeded and redundant with “given topic”.**

 This sentence has been edited accordingly.

**Page 3: “for a gene with ± 1000 associated articles and five participants”. This phrase is too concise to have meaning, as a gene cannot have participants.**

 We have edited this sentence to read:

 “For instance, for a gene with about 1,000 associated articles, five participants could work together on this same gene, each focusing on a different theme.”

**Page 4, Step 1. No mention is made of NCBI’s Advanced Search Builder; MeSH terms; or truncation by asterisk. The manuscript should explain why these advanced features are not taught.**

 Indeed, thanks for the suggestion. We have re-written this section and mention the availability of the advanced search builder and provided the corresponding URL. We also pointed to training material available on the NCBI website. In our experience truncation by asterisk can produce unwanted results and increase false positives, also did not want to recommend using it in this case.

**Page 4, bottom: “The field restriction tag [tw] (or alternatively the more restrictive [tiab]) should be added after each term… to limit the search to titles and abstracts”. Actually, it is [tiab] that limits the search to titles and abstracts. The broad [tw] searches almost everything (see https://pubmed.ncbi.nlm.nih.gov/help/#tw).**

 Yes, this is correct, and this entire section has been rewritten for clarity. We found the [tw] field to be effective in preventing PubMed from trying best guesses when few entries are returned. [tiab] can be used when users wish to further restrict the search. But both should provide good results for our purpose.

**Page 5: “examples provided above”. Where? Vague cross-references like this make reading difficult.**

 Thanks for pointing this out. We are now providing those examples instead.

**Page 6: “For instance, when assessing the novelty …”. This incomplete sentence is lacking a subject-verb pair. Similar problem on page 13, bottom.**

 This has been corrected.

**Page 6, bottom: “The Boolean argument AND is added”. AND is a Boolean operator, not argument.**

 Indeed, this has been corrected.

**Page 6, bottom: ‘… AND (“Liver cancer”[tw] OR “Liver carcinoma”[tw] OR “Hepatic carcinoma”[tw], etc.)’. A query cannot contain “, etc.”. It should be removed, and a final period added to the sentence.**

 This has been corrected as well.

**Page 9, Step 5. This part is excessively wordy and suggests an uneasiness with the concepts. We read “is the measure by which productivity is measured”, “it can be at first important to be able to identify information that is worth capturing” and “A specific topic would be selected as the focus … to limit the amount of literature that would need to be covered”.**

 We rewrote and condensed this paragraph.

**Page 13: Webex. Not WebEx.**

 This has been corrected.

**Punctuation**

 Thank you, that was very useful, and we have now made the necessary edits throughout.

- **Comma after “Supporting material” in the third paragraph of Introduction. Comma after “This is a skill that is worth developing” (page 9).**
- **Replace ± with “about” (first paragraph of Methods).**
- **Hyphens: no hyphen after -ly adverbs, e.g. “early career researcher”. Use hyphens in “gene-associated literature”, “false-positive result” and “false-negative result” (but not in “false positives” or “false negatives”).**
- **There are many instances where the required space between words is missing, especially when a quotation mark is used, e.g. “For example, under’Human diseases and … associated keywords could be’liver cancer’,’hepatic carcinoma’, …” (page 5). The entire manuscript should be checked and spaces added where needed. A hint is that the curly quotation mark is in the wrong direction before the word when a space is missing.**
- **Double quotation marks should be used in all cases except when nesting is needed. Nesting should be used on page 5 where the sentence: ' *This was rectified by adding the AND “cross-reactive protein [tw] argument to each of the ambiguous acronym.' *should be changed to: ' *This was rectified by adding the ‘AND “cross-reactive protein [tw]”’ argument to each of the ambiguous acronyms.' *or more simply: ' *This was rectified by adding ‘AND “cross-reactive protein [tw]”’ to each of the ambiguous acronyms.'***

# Peer review: Reviewer response for version 1

**DOI:** 10.5256/f1000research.39457.r86220

**Published:** 2021-06-15

**Authors:** Gary S. McDowell

**Affiliations:** 1 Lightoller LLC, Chicago, IL, USA

The authors discuss the need and provide a curriculum for early career researchers to develop the ability to survey and synthesize the background literature to a field in an appropriate manner. This is demonstrated with publicly available “omics” data as an example.

 This article gives an excellent description of a training workflow that is suitable and adaptable for training in systematic literature profiling. The article provides clear illustrative use case examples that are very helpful in demonstrating the results of the training workflow, and provide excellent materials for others to work from in developing similar materials.

Is the rationale for developing the new method (or application) clearly explained?

Yes

Is the description of the method technically sound?

Yes

Are the conclusions about the method and its performance adequately supported by the findings presented in the article?

Yes

If any results are presented, are all the source data underlying the results available to ensure full reproducibility?

Yes

Are sufficient details provided to allow replication of the method development and its use by others?

Yes

Reviewer Expertise:

Early career researcher experiences, training and professional development.

I confirm that I have read this submission and believe that I have an appropriate level of expertise to confirm that it is of an acceptable scientific standard.
